# Millimeter-Wave Chemical Sensor Using Substrate-Integrated-Waveguide Cavity

**DOI:** 10.3390/s16111829

**Published:** 2016-10-31

**Authors:** Muhammad Usman Memon, Sungjoon Lim

**Affiliations:** School of Electrical and Electronics Engineering, Chung-Ang University, 84 Heukseok-ro, Dongjak-gu, Seoul 156-756, Korea; musmanm@outlook.com

**Keywords:** SIW, fluidics, chemical sensor, multilayer cavity, ethanol

## Abstract

This research proposes a substrate-integrated waveguide (SIW) cavity sensor to detect several chemicals using the millimeter-wave frequency range. The frequency response of the presented SIW sensor is switched by filling a very small quantity of chemical inside of the fluidic channel, which also causes a difference in the effective permittivity. The fluidic channel on this structure is either empty or filled with a chemical; when it is empty the structure resonates at 17.08 GHz. There is always a different resonant frequency when any chemical is injected into the fluidic channel. The maximum amount of chemical after injection is held in the center of the SIW structure, which has the maximum magnitude of the electric field distribution. Thus, the objective of sensing chemicals in this research is achieved by perturbing the electric fields of the SIW structure.

## 1. Introduction

In the modern era, chemicals in the fluidic state have been used in diverse industrial applications. These fluidic chemicals are normally classified and stocked as per policies of globally harmonized systems (GHS) which have regulations for labeling and categorization of fluidic chemicals, and also the Material Safety Data Sheet (MSDS). However, unlabeled or non-categorized liquid fluids frequently occur in experimentations. Quite a few of these liquids are unsafe for human health and the body. For instance, methyl alcohol is injurious to the human nervous system and can be a cause of severe medical conditions such as coma, blindness or immediate death if it is inhaled [1]. Consequently, the exact quantification or detection of chemical fluids used in many industrial applications is very important.

For analyzing chemicals or manipulating them using infinitesimal quantities, we have microfluidics as a valuable tool and the proposed method for applications such as analyses of blood and bioassays, and for monitoring industrial value or quality of service [2,3,4,5,6]. Previously, the methods engaged for examining bioassays and assessing liquid quality required huge volumes of fluidics to fill the valves or the tubing [7,8]. A large quantity of those liquids was unused, discarded and never used for experimental needs. For eradicating this issue of wasting unused fluidic chemicals, microfluidic arrangements were announced. Analysis of the chemicals or fluids can now be achieved on absolutely small volumes, normally at the microliter or nanoliter range of liquids. It is very promising due to the colossal combination of detection and interface electronics, fluidic management geometries, and micrometer-sized fluidic channels on a single chip [6]. Recent advancements in waveguides have enabled miniaturization and integration with optical, fluidic and electronic components in a chip for chemical sensor technology [9,10,11]. Some of the newly addressed liquid-integrated RF (radio frequency) structures are using fluids as a new and reusable dielectric material for antennas, RF resonators or transmission lines [12,13,14].

**Figure 1:**
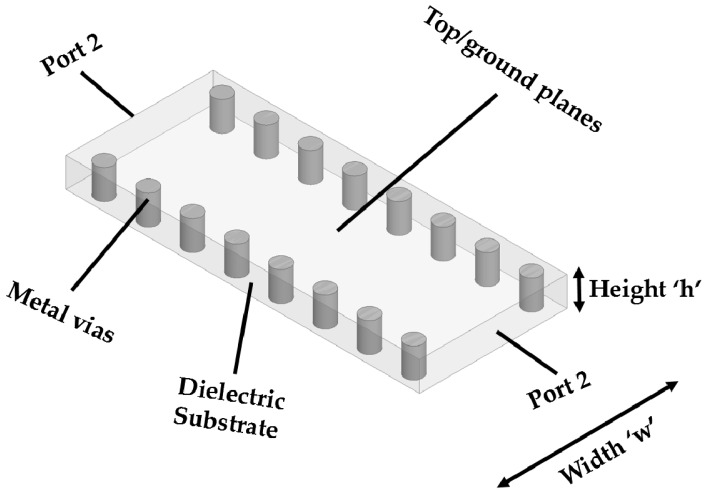
Illustration of SIW structure.

A substrate-integrated waveguide (SIW) structure is illustrated in Figure 1; the SIW is a promising candidate for advancements in the planar RF structures used for applications in wireless systems and many more [15,16,17,18,19,20,21]. Figure 1 shows that the SIW construction contains two rows of vias (cylindrical conductor) joining the top and bottom plane, and in between the structure is sealed with any low-loss dielectric material [22]. SIW components have been extensively utilized due to their easy integration and simple fabrication, coupled to the advantages of a planar printed circuit board (PCB) and metallic waveguide. As relates to the microstrip lines (MSLs) and coplanar lines, SIW components are easy to handle, cost-effective, simple in fabrication, compact and lighter. SIW geometries also have the properties of classic metal waveguides, i.e., the maximum quality factor (QF), lowest losses, proper shielding and all-out power-handling ability. The biggest advantage of this waveguide structure is that it allows you to fabricate a complete circuit in a planar formation of coalescing circuits, transitions, four-sided waveguides and planar antenna configurations by means of classic PCB technology. Additionally, SIWs also allow you to install one or more than one chipset on the same structure material, so it can easily be incorporated with different systems having different parameters, also decreasing overall losses. System-on-substrate (SoS) is an excellent candidate for evolving high-performance, easy-to-fabricate and cost-effective mm-wave (millimeter) elements. Lately, some significant works have been reported in the literature towards the study of the miniaturization of SIW technology [23]. This widespread research drive has led to the improvement of exclusive design approaches for SIW contrivances, and some new procedures and techniques have also been proposed to make it more efficient and reusable [24].

Polydimethylsiloxane (PDMS), a commercial silicone elastomer with a low Young’s modulus (<2 MPa), has been routinely used for constructing fluidic geometries [25]. It is very flexible and contains a low surface energy and low modulus; these assets permit it to be adaptive in attaching to surfaces easily. PDMS has a low dielectric constant (2.67) and a high loss tangent (0.0375) [26]. As discussed above, RF components also allow the integration of fluidic channels for different sensing applications. A microwave and fluidic resonator sensor was presented in [13]. The advantage of a fluidic channel is that it creates a convenient environment to gather measurement data from a nanoliter amount of liquid. This implies that the resonant frequency of an RF circuit can be switched and sensed by the injection of a nanoliter liquid.

In our research, a non-contact fluidic SIW cavity resonator is proposed for chemical sensing. The initial structure is designed using substrate-integrated waveguide (SIW) technology, which provides various advantages: (1) low loss; (2) a high quality (Q)-factor; and (3) a light weight, as mentioned earlier [22]. In our proposed research, a fluidic channel is constructed on the PDMS slab, which is then incorporated between two layers of the SIW on the top and bottom to achieve a higher frequency-shifting ability. Hence, the resonant frequency of the structure is widely influenced by the injection of a very small amount of liquid sustained at the center position of the SIW inside the fluidic channel. The microwave cavity structure can be seen in the literature [27].

## 2. SIW Sensor Design

**Figure 2:**
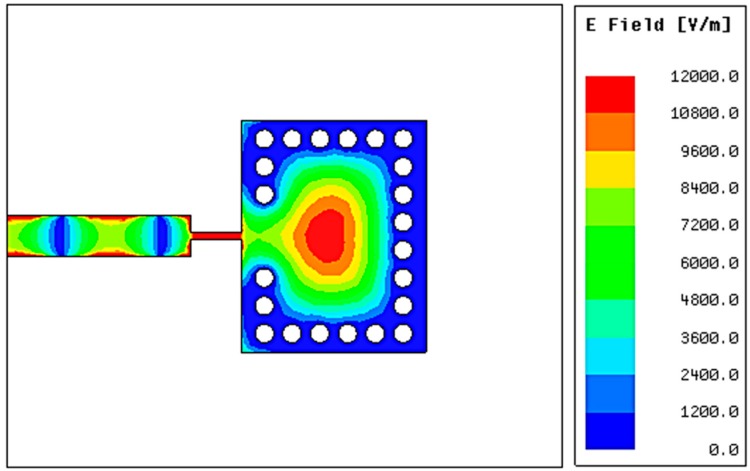
Electric field distribution of a SIW cavity resonator.

**Figure 3:**
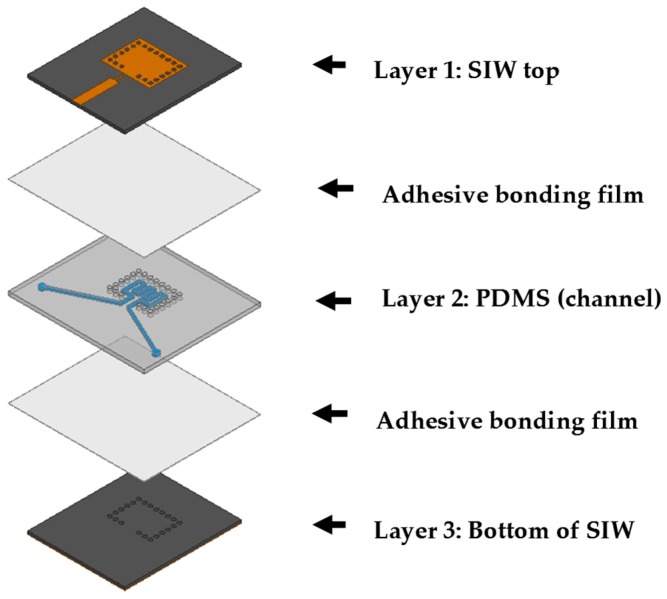
Layers of the proposed SIW chemical sensor.

**Figure 4:**
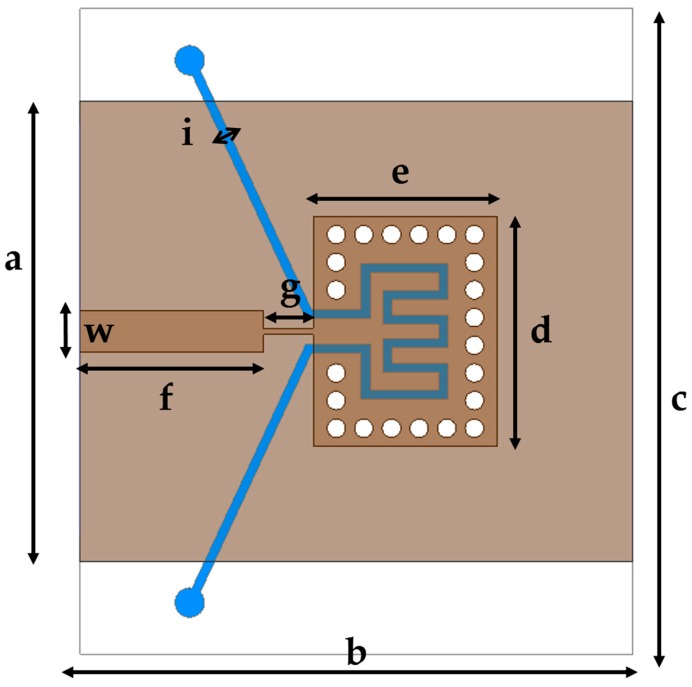
Proposed SIW chemical sensor (top view).

SIW planar structures are simple in fabrication and can be cascaded with millimeter-wave and microwave-integrated circuits (ICs) as printed circuit boards (PCBs). Electric field distribution of a SIW cavity structure is given in Figure 2. It is clearly seen from Figure 2 that the highest magnitude of the electric field is concentrated towards its center. In this work, the SIW cavity resonator is used because of its easy fabrication, low parasitic loss, and high Q factor [28,29]. Its resonant frequency is dependent on the effective dielectric constant of a substrate. In addition, its effective dielectric constant can be changed by injecting chemicals inside the SIW cavity. Therefore, the proposed SIW sensor can detect the dielectric constant of different chemicals from the variation of the resonant frequency. The layers of the proposed SIW chemical sensor are shown in Figure 3. A fluidic channel is constructed on the middle layer (PDMS slab of 1 mm thickness) at a location which corresponds to the center of the SIW (extreme e-field). The proposed structure has three layers, as shown in Figure 3; two of them (top and bottom) are built on the substrate of RT/Duroid 5870, which has a permittivity (ε_r_) of 2.33 and a height *h* of 0.79 mm. In order to bond three layers, two bonding layers are used. When the height *h* is way smaller than the width *W_SIW_* and length *L_SIW_*, the dominant mode of the operating frequency of the SIW cavity is given as:

(1) $$f_{mn}=\frac{1}{2\pi \sqrt{\mu \varepsilon }}\sqrt{{\left(\frac{m\pi }{W_{SIW}}\right)}^{2}+{\left(\frac{n\pi }{L_{SIW}}\right)}^{2}}$$

where ε is the permittivity, µ is the permeability of the dielectric material, *m* and *n* are the modes of the SIW cavity. The designed SIW is illustrated in Figure 4 and its geometrical parameters are given in Table 1.

As seen in Figure 4, the PDMS layer is a sandwich between two Duroid layers and it has a fluidic channel (E-shaped) constructed so as to occupy the maximum possible liquid towards the center of the SIW cavity. The width and depth of the fluidic channel are kept to 0.5 mm. The E-shaped channel design in the center is chosen to avoid air gaps inside the channel (achieving complete sustainability) and the width of the channel is maintained the same throughout for smooth flow of the liquids inside the channel. Several chemicals are injected into the fluidic channel and their respective frequency responses are recorded.

## 3. Simulation Results

**Figure 5:**
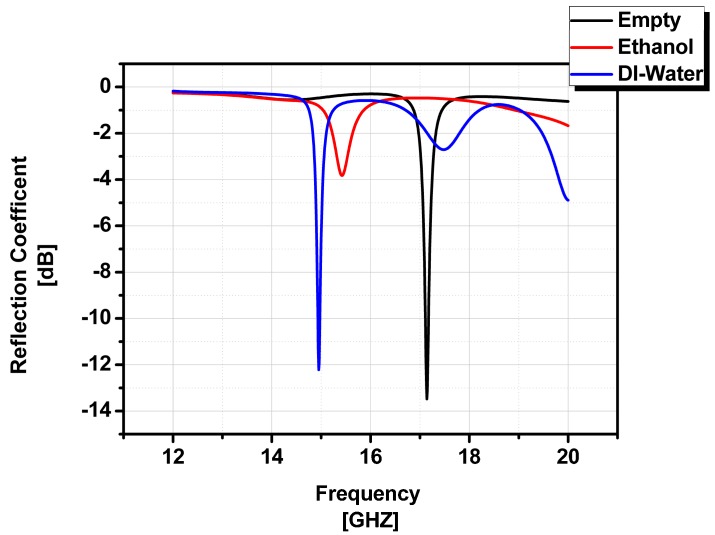
Simulated reflection coefficients of ethanol-filled, DI-water-filled and an empty state of microfluidic channel.

A full-wave simulation was carried out using an HFSS (high frequency structure simulator). The PDMS sheet of 1 mm thickness is characterized and its electrical properties are used to define a material in the HFSS. Ethanol and DI (deionized) water are also characterized at 18 GHz frequency and are used to fill the fluidic channel in order to validate our design and idea of sensing chemicals in the millimeter-wave frequency range. The simulated reflection coefficients of the ethanol and DI-water-filled chemicals and their distinct frequency responses are plotted in Figure 5.

Figure 5 shows three different frequency responses. When the channel is empty (air only), the resonant frequency is 17.08 GHz. When the channel is filled with DI water and ethanol, the resonant frequency is changed to 14.95 GHz and 15.56 GHz, respectively. Therefore, it is expected that this structure can work as a chemical sensor to detect several chemicals with different permittivity.

## 4. Experimental Demonstration

**Figure 6:**
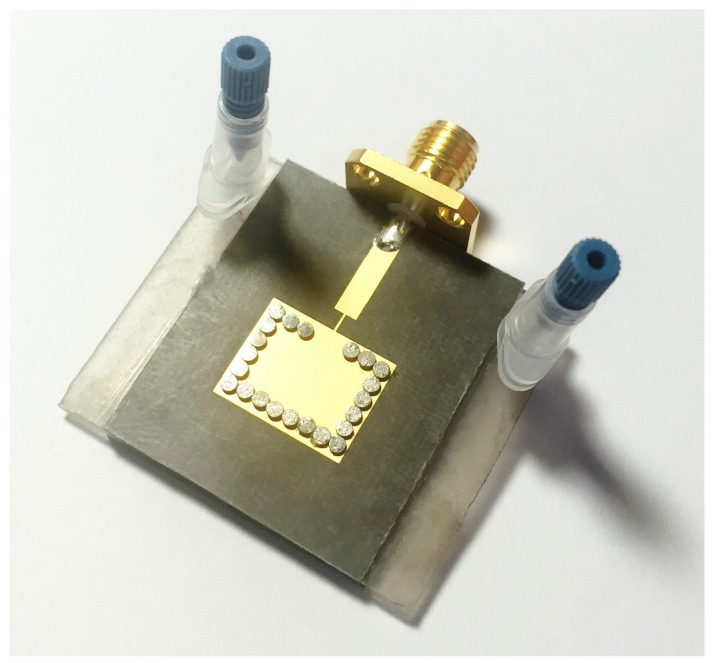
Photograph of the fabricated prototype.

Figure 6 is a snapshot of the proposed sensor prototype. The top and bottom layers are fabricated using a PCB etching process, and the middle layer PDMS (with the fluidic channel) is made using a laser etching machine. In order to bond the Duroid 5870 substrates and PDMS, we used ARcare^®^92848 manufactured by Adhesives Research, Inc. (Pennsylvania, PA, USA) for the adhesive bonding film. This film is used in biosensor spacers and general diagnostic device bonding. After bonding all layers, we drilled holes in order to make metallic vias. Next, silver pins are inserted into the empty holes. Finally, pins are connected to the top and bottom conductive patterns of the Duroid substrate by soldering them.

**Figure 7:**
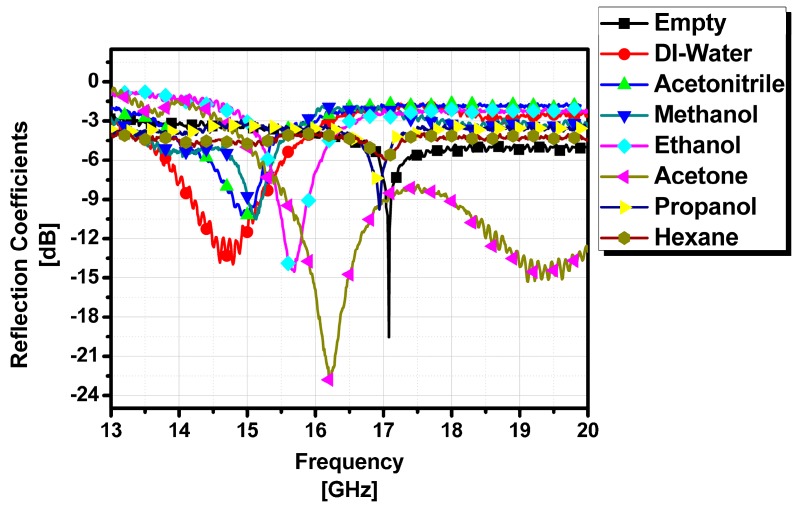
Measured reflection coefficients.

Two inlet/outlet ports are installed to simplify the injection of the chemicals into the fluidic channel of the middle layer (PDMS). One is used as an inlet and another is used as an outlet, confirming that the chemical has completely filled the fluidic channel. Seven different chemicals are injected one by one in the fluidic channel and the frequency responses are recorded using an HP 8510C VNA (Vector Network Analyzer) (Hewlett Packard (HP), Palo Alto, CA, USA). Dielectric constants of seven chemicals (DI water, acetonitrile, methanol, ethanol, acetone, propanol and hexane) in 13–18 GHz are extracted from the measurement results as listed in Table 2. Because their dielectric constants are different, it is expected that their resonant frequencies must be different each other. If the effective dielectric constants of the chemicals are same, it is difficult to detect the chemicals, which is a limitation of the proposed idea. After each chemical’s response is measured, the sample is drained completely from the channel to avoid cross-contamination occurring between any two chemicals. The measured reflection coefficients of the seven chemicals are plotted in Figure 7. Because each chemical has a different dielectric constant, it gives a different resonant frequency.

**Figure 8:**
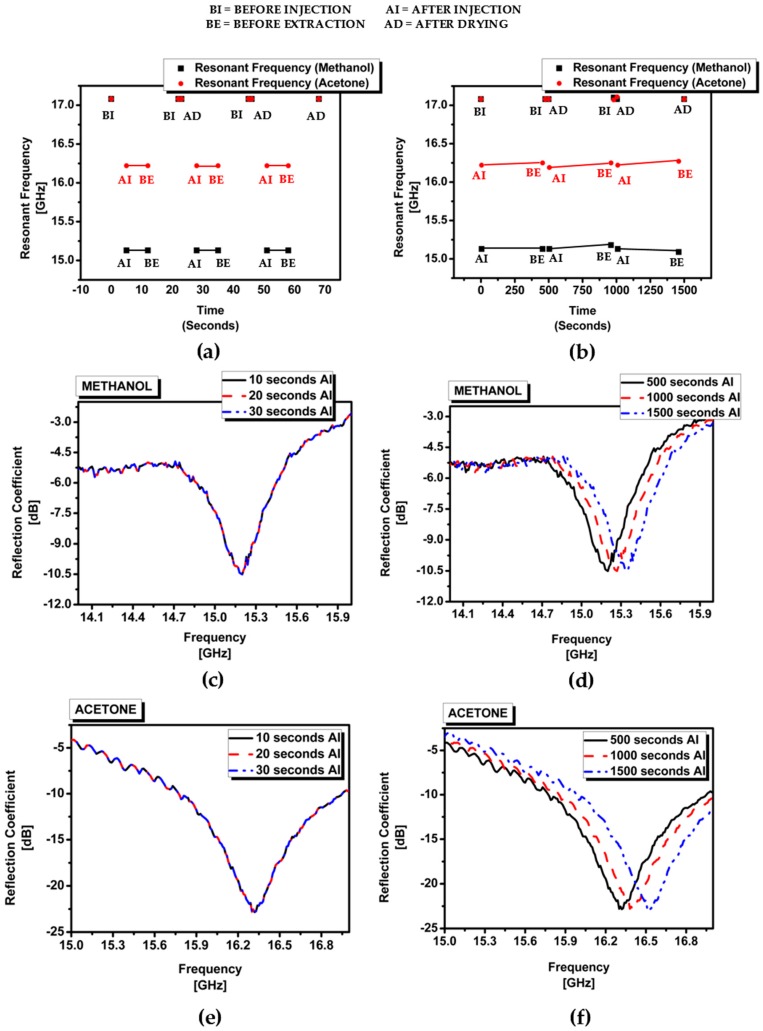
Repeatability test of the proposed SIW chemical sensor for methanol and acetone: (**a**) frequency variation of test #1 (fast injection and extraction process); (**b**) frequency variation of test #2 (slow injection and extraction process); (**c**) measured reflection coefficients of test #1 with methanol; (**d**) measured reflection coefficients of test #2 with methanol; (**e**) measured reflection coefficients of test #1 with acetone; (**f**) measured reflection coefficients of test #2 with acetone.

It is reported that low-solubility solvents can be used with PDMS without swelling [30]. In addition, the swelling ratio is dependent on the solvent vapor exposure time [31]. Therefore, the repeatability of the proposed sensor is experimentally demonstrated by sequentially injecting, extracting and drying it five times. Fast and slow processes are performed with methanol and acetone as test #1 and #2, respectively. Figure 8a shows the resonant frequency variation at each time point for test #1. After injecting methanol or acetone in the channel of the chemical sensor, we extracted the chemical from the channel after 10 s. We dried the empty channel for 20 s using hot air. We performed this sequence five times. In the fast process with both methanol and acetone, the resonant frequency at the empty channel was 17.15 GHz and it did not change, although the channel was reused, as shown in Figure 8a. In addition, as shown Figure 8c,e, the resonant frequencies at the methanol- and acetone-filled channels were not changed, although repeatedly tested. Figure 8b shows the resonant frequency variation at each time for test #2. After injecting methanol or acetone in the channel of the chemical sensor, we extracted the chemical from the channel after 500 s. It is observed from Figure 8b that the resonant frequency with methanol was changed from 15.17 to 15.28 GHz after 500 s. Similarly, the resonant frequency with acetone was changed from 16.24 to 16.37 GHz after 500 s. Therefore, the proposed sensor is unreliable in the slow process. After extracting the chemical, we dried the empty channel for 20 s using hot air. In the second sequence, the resonant frequency with the empty channel was changed to 17.11 GHz while the resonant frequency was 17.15 GHz in the first sequence. It is observed from Figure 8d that the resonant frequencies with the methanol-filled channel were 15.17, 15.28, and 15.40 GHz for the first, second, and third sequence of the slow process. It is observed from Figure 8f that the resonant frequencies with the acetone-filled channel were 16.24, 16.37, and 16.52 GHz for the first, second, and third sequence of the slow process. Therefore, the proposed chemical sensor can be repeatedly used when chemicals are extracted in 200 s after injection. The measurement results validated the sensing mechanism successfully and it is demonstrated from the results that our proposed SIW chemical sensor is non-contact, it can detect several chemicals at the millimeter-wave frequency range and it can also be re-used multiple times. Chemical identification and purity determinations can be done much better with ESI mass spectrometry or colorimetric tests [32,33]. Nevertheless, the proposed RF technology still has benefits because the proposed chemical sensor is non-contact and reusable. The fabrication process is much simpler than other non-RF chemical sensors.

**Figure 9:**
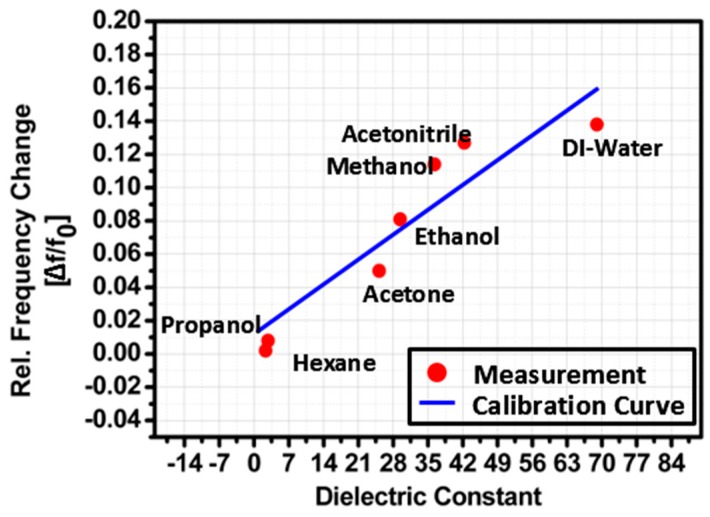
Sensitivity of the proposed SIW chemical sensor.

In Figure 9, the measured relative frequency change (Δ*f*/*f*_0_) is plotted at the dielectric constant (ε_r_) of each chemical. In order to see the linearity of the proposed sensor, the calibration curve is plotted. It is observed from Figure 9 that the relationship between Δ*f*/*f*_0_ and ε_r_ is close to *y* = 0.0023*x* + 0.0076 from ε_r_ = 2.3 to 79. Linearity cannot be satisfied in all chemicals because the frequency is not linearly proportional to the dielectric constant, as shown in Equation (1). When sensitivity S_ε_ is defined by the slope angle of the calibration curve, it is 0.0023 mm^−1^.

(2) $$S_{\varepsilon }=\frac{\delta \left(\Delta f/f_{0}\right)}{\delta \left(\varepsilon_{r}\right)}\;[{\text{mm}}^{-1}],$$

where ∆*f* is the frequency shift between an empty state and a chemical-filled state (∆*f* = *f*_empty_ − *f*_chemical_); *f*_0_ is the frequency at the empty channel state; and ε_r_ is the characterized dielectric material of the chemical in Table 2.

## 5. Conclusions

It is successfully demonstrated that the resonant frequency of a SIW structure is influenced enormously when any of the chemical is injected in the fluidic channel on the PDMS layer of the structure. It makes the proposed millimeter-wave sensor highly sensitive, non-contact and reusable, and it is also compact in size. The feasibility of the proposed sensor is numerically and experimentally demonstrated in 14 to 18 GHz. Nanofabrication technology enables the system size to be much smaller by increasing the operating frequency to terahertz bands. Therefore, the proposed idea can be commercially used as a chemical sensor for chemistry laboratory applications where the identification of chemicals is required to manage the chemical shelves or categorize them with respect to their level of hazard.

## Figures and Tables

**Table 1:** Design parameters of the proposed SIW chemical sensor.

Parameter	Dimension (mm)	Parameter	Dimension (mm)
a (also *W*_SIW_)	25	f	9.95
b	30	g	2.7
c	35	i	0.5
d	12.5	w	2.24
e (also *L*_SIW_)	10		

**Table 2:** Extracted dielectric constants of chemicals in 13–18 GHz.

No.	Chemicals	Dielectric Constant (ε_r_)
1	DI Water	79
2	Acetonitrile	42.3
3	Methanol	36.3
4	Ethanol	29.4
5	Acetone	25.2
6	Propanol	2.8
7	Hexane	2.3
