# Correction: Lim, S., et al. Millimeter-Wave Chemical Sensor Using Substrate-Integrated-Waveguide Cavity. *Sensors* 2016, *16*, 1829

**DOI:** 10.3390/s17010029

**Published:** 2016-12-24

**Authors:** Muhammad Usman Memon, Sungjoon Lim

**Affiliations:** School of Electrical and Electronics Engineering, Chung-Ang University, 84 Heukseok-ro, Dongjak-gu, Seoul 156-756, Korea; musmanm@outlook.com

We wish to make the following corrections to their paper [1]:

The title of the paper ‘Millimeter-Wave Chemical Sensor Using Substrate-Integrated-Waveguide Cavity’ should be changed to ‘Microwave Chemical Sensor Using Substrate-Integrated-Waveguide Cavity’.

In the second line of the abstract, on the fifth line of Section 3 (Simulation Results), and in Section 4 (Experimental Demonstration) on Page 7, the phrase ‘millimeter-wave frequency range’, should be changed to ‘microwave frequency range’. Also, on the third line of the Conclusion Section, ‘millimeter-wave’, should be replaced with ‘microwave’. 

It is well-known that millimeter-wave corresponds to a frequency of over 30 GHz. As the proposed sensor functions at around 18 GHz, it should be called ‘microwave’. We apologize for any inconvenience these changes have caused to readers. The changes do not affect the scientific results. The manuscript will be updated and the original will remain online on the article webpage.
